# *Mycobacterium haemophilum* as the Initial Presentation of a B-Cell Lymphoma in a Liver Transplant Patient

**DOI:** 10.1155/2014/742978

**Published:** 2014-01-12

**Authors:** T. Doherty, M. Lynn, A. Cavazza, E. Sames, R. Hughes

**Affiliations:** Ashford and St. Peters NHS Trust, Chertsey, Surrey KT16 0PZ, UK

## Abstract

A 66-year-old woman presented with pustular lesions of her face, trunk, and limbs and an acute arthritis of the knees and elbows. She had a complex medical background and had been on immunosuppressants for three years after a liver transplant. Tissue samples from her skin lesions and synovial fluid showed acid-fast bacilli. *Mycobacterium haemophilum*, an atypical mycobacteria, was later grown on culture. During her treatment with combination antibiotic therapy, she developed a pronounced generalised lymphadenopathy. Histology showed features of a diffuse B-cell lymphoma, a posttransplant lymphoproliferative disorder (PTLD).

## 1. Presentation

**Figure 1:**
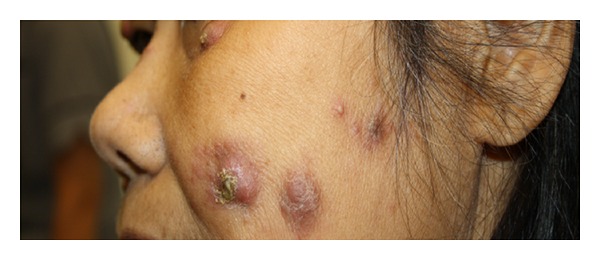


**Figure 2:**
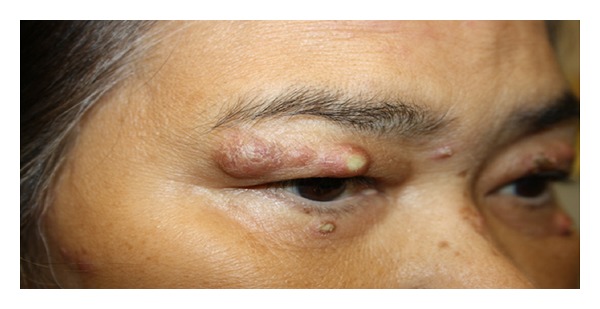


In October 2012 a 66-year-old, Filipino, retired psychiatric nurse attended the rheumatology department in a wheelchair. She gave a 3-week history of painful and swollen knees, ankles, and elbows. Walking had become difficult. Systemically she was unwell and described weight loss (five kilograms over a month), malaise, and anorexia. Widespread tender and raised pustular skin lesions (Figures 1 and 2) were noted over her face, arms, chest, and legs. She reported that these were itchy and painful and had failed to improve with a two-week course of oral flucloxacillin (500 mg QDS). Further examination revealed that she was clinically anaemic and confirmed a large joint polyarthritis with bilateral knee effusions. Other systems examination was unremarkable.

## 2. Past Medical History and Medications

Her significant medical history included a liver transplant, in 2009, for a primary malignant neoplasm (hepatocellular carcinoma).

Her other past medical history included idiopathic thrombocytopenic purpura, type 2 diabetes (diet controlled), chronic kidney disease (stage 3), vitamin B12 deficiency, a hysterectomy (for menorrhagia), and secondary osteoporosis due to steroids.

As part of her antirejection regime she had been taking mycophenolate mofetil (2 grams daily) and low dose prednisolone (5 milligrams daily).

She also took alendronic acid (70 mg weekly) and calcium carbonate/cholecalciferol (1.5 g/400 IU daily). For her recent joint pains she had been using paracetamol and codeine.

## 3. Investigations

Initial blood tests showed a Hb of 7.0 g/dL (hypochromic and microcytic), CRP 168 mg/L, WCC 9.6 × 10^9^/L, neutrophils 9.0 × 10^9^/L, urea 10 mmol/L, and creatinine 122 *μ*mol/L.

She was admitted to hospital and transfused with three units of blood.

Investigations included an upper gastrointestinal endoscopy which revealed multiple shallow ulcers and positive *Helicobacter pylori* urease test. Her left knee aspirate showed scanty positively birefringent crystals on microscopy and pseudomonas on Gram staining. A mid-stream urine sample grew *E. coli*. Electrocardiograms and a chest X-ray were normal. CT of her chest, abdomen, and pelvis was reported as normal aside from a left upper kidney pole abnormality, which was likely to represent a cyst. This was later reviewed by the urologists who were unconcerned but arranged followup.

## 4. Initial Management and Further Microbiology Results

For her septic arthritis and her skin infection she was commenced on intravenous meropenem (1 gram TDS). She received eradication treatment for the *Helicobacter* (one week of twice-daily omeprazole 20 mg, metronidazole 400 mg, and clarithromycin 250 mg). Further microbiology results from a skin lesion biopsy and a knee synovial aspirate both showed *Pseudomonas* bacilli and stained strongly positive for acid-fast bacilli (AFB). Samples of synovial fluid from the knee were sent to the National Tuberculosis Reference Laboratory in London for further characterization. This showed that the AFB were *Mycobacterium tuberculosis* (MTB) complex negative indicating an atypical mycobacterium. Culture results followed showing a rare atypical AFB, *M. haemophilum*.

She was initiated on the recommended regime of a combination of oral ciprofloxacin (500 mg BD), clarithromycin (500 mg BD), and rifampicin (300 mg BD) and discharged home. On this combination she had an initial, nonsustained response (of one month) in terms of her skin lesions and joints, but she relapsed requiring rehospitalisation. She was then commenced on a combination of oral clarithromycin 500 mg BD and intravenous imipenem QDS which led to clinical improvements. She was discharged home with the community team administering her antibiotics.

## 5. Complications

Over the subsequent months she was reviewed on a monthly basis in the rheumatology outpatients department. At her review in February 2013, she had a palpable right preauricular (1 cm) and a left groin lymph node (1.5 cm).

A repeat CT of her chest, abdomen, and pelvis showed new subcutaneous lymphadenopathy in the anterior abdominal wall and in the flanks at the level of her fifth lumbar vertebra. An enlarging node was noted in the left ischiorectal fossa and there were two abnormal nodes in the right hemipelvis.

Histology of the left groin node showed lymph node tissue containing medium-to-large sized lymphocytes with a few inflammatory cells and small spotty foci of necrosis with a few neutrophil polymorphs in keeping with a high grade lymphoma.

Immunohistochemistry was positively staining for CD20, CD79a, bcl-2, CD30 (focal), and bcl-6 (focal). There was lambda restriction, T cell markers showed reactive T cells, and MIB-1 was increased (60% expression), all consistent with a diffuse large B-cell lymphoma.

She went on to have a positron electron tomography (PET) scan which also correlated with a diagnosis of lymphoma.

She was promptly seen by the haematologists who made the decision to treat her lymphoma with rituximab. We maintained regular outpatient followup and continued her antibiotics for her skin lesions which appeared to worsen with any attempt to withdraw from the regime.

Eleven months on from her initial presentation she is clinically well. She has residual skin lesions (eighty percent improvement) and she has completed rituximab treatment. A reduction in her mycophenolate to 500 mg BD was sanctioned by her liver specialist and we intend to complete twelve months of her current antibiotics before stopping (10 months completed).

## 6. Discussion

Human *M. haemophilum* infection was first diagnosed by Sompolinsky in 1978. This first description of the infection manifested as cutaneous lesions in a 51-year-old female with Hodgkin's lymphoma [1].

Since this report, there have been over 220 published cases of human *M. haemophilum* infecting mainly immune-compromised adults and healthy children. The most recent review was done by Lindeboom et al. in 2011 [2]. The adult infections most frequently involve the skin, but there are a number of case reports with septic arthritis [3], disseminated [4] and pulmonary infections [5], pyomyositis [6], osteomyelitis [7], and ophthalmic involvement [8, 9]. Rarely, the involvement of other systems has been reported. There has been one case report of an epididymal abscess in a patient with a renal transplant [10] and central nervous system involvement in two patients [11].

Immunocompetent adults have been known to acquire cutaneous infections transmitted in permanent makeup or tattoo ink [12–14]. In children, cervicofacial lymphadenitis is the most common manifestation and is not associated with immunocompromise [15].

In veterinary medicine *M. haemophilum* has been isolated in zebra fish and useful epidemiology studies have been conducted in reservoirs. Studies have concluded that identical mycobacterium strains have been found infecting fish and in the biofilms, but more research is needed [16]. In 2002 Hernandez-Divers et al. published a case of a royal python with pulmonary mycobacteriosis due to dual infections of *M. haemophilum* and *M. marinum* [17]. Pai et al. in 2003 isolated *M. haemophilum* from hospital cockroaches in China and speculated on their role in transmitting the infection [18]. In 2006 Jacob et al. reported a case of a *M. haemophilum* epidural granuloma in an American bison [19].

Despite the case reports, it is an infection that may be underdiagnosed. The reasons are its unusual culture requirements of an iron enriched (ferric ammonium citrate or hemin) medium and the low incubation temperatures of 30 to 32°C for growth *in vitro*. In their review in 1996, Saubolle et al. recommended requesting such cultures in immunocompromised patients with septic arthritis or skin lesions, bone marrow transplant patients with pulmonary lesions, adenitis in children and in situations where acid-fast strains from previous specimens were acid-fast positive, but the cultures were negative [20].

The natural habitat and the mode of transmission remain unclear. There has been speculation that contaminated water sources have borne infection in humans and fish, although no clinical isolates have been directly linked to environmental isolates. The virulence is considered low, mainly based on animal studies where healthy mice and guinea pigs were inoculated with large volumes of bacilli (intravenously, subcutaneously and intramuscularly) and suffered no ill effects. However mice who were given prednisolone developed ear lesions with AFBs similar to cutaneous lesions seen in human adults [21].

The treatment can be complex and prolonged and removing the cause of immunosuppression is advocated [22]. In Lindeboom et al.'s review they described 33 cutaneous infections with *M. haemophilum* in immunocompromised adults [2]. Antibiotic regimens usually included clarithromycin and were most commonly inclusive of ciprofloxacin and rifampicin. Other antibiotics used were doxycycline, ethambutol, rifabutin, isoniazid, cycloserine, amikacin, azithromycin, trimethoprim-sulfamethoxazole, pyrazinamide, minocycline, gatifloxacin, and levofloxacin. The duration of the antibiotic treatments varied from one to forty-two months. None of the regimens for cutaneous lesions included imipenem, but amongst twelve patients with disseminated disease, one cardiac transplant patient with skin, pulmonary and joint involvement was successfully treated with a combination of imipenem, clarithromycin, ciprofloxacin, and doxycycline.

This lady's diagnosis was delayed and she failed to sustain a response to the most commonly accepted regimens and those antibiotic recommended by the National Tuberculosis Centre. Unfortunately there is no evidence based approach for an alternative regime, but we continued to rely on the antibiotic regimen which appeared to have generated a sustained response. Therefore we continued intravenous imipenem (QDS) and per oral clarithromycin (BD).

This lady had several risk factors for the development of *M. haemophilum* including her background of a liver transplant and subsequent immunosuppression with prednisolone and mycophenolate. Her vulnerability was enhanced by her diabetes mellitus and the underlying undiagnosed B-cell lymphoma. She continues to make significant improvements on the protracted course of antibiotics, the treatment of her lymphoma, and the reduction of her mycophenolate dose.

The responses to antibiotics are not uniform, but several authors have published reports of success with prolonged combination therapy. Seeking advice from the National Tuberculosis Referencing Centres is recommended, but therapy should be measured against clinical responses.

This is the first case report of *M. haemophilum* preceeding a diagnosis of lymphoma. Our case is recognisably complex requiring multiple diagnostic steps with specialist input from rheumatology, dermatology, microbiology, haematology, hepatology, and community healthcare teams.

A number of case reports of *Mycobacterium haemophilum* in patients with an underlying rheumatological diagnosis have also been published. The first case report of a patient with a diagnosis of lupus treated with mycophenolate before developing the infection was published in 2002 [23].

Patients on biologic treatments for inflammatory arthritis have also developed *M. haemophilum* infections [24]. We support Saubolle et al.'s recommendation for iron enriched low temperature cultures of synovial fluids or skin biopsies in immunocompromised patients. This case has served to increase our awareness of this infection in patients presenting to the rheumatology with acute arthritides and those patients under rheumatology followup on immunosuppressants.

## References

[1] Sompolinsky D, Lagziel A, Naveh D, Yankilevitz T (1978). Mycobacterium haemophilum sp. nov., a new pathogen of humans. *International Journal of Systematic Bacteriology*.

[2] Lindeboom JA, van Coppenraet LESB, van Soolingen D, Prins JM, Kuijper EJ (2011). Clinical manifestations, diagnosis, and treatment of Mycobacterium haemophilum infections. *Clinical Microbiology Reviews*.

[3] Olsen RJ, Cernoch PL, Land GA (2006). Mycobacterial synovitis caused by slow-growing nonchromogenic species: eighteen cases and a review of the literature. *Archives of Pathology and Laboratory Medicine*.

[4] Kelley CF, Armstrong WS, Eaton ME (2011). Disseminated *Mycobacterium haemophilum* infection. *The Lancet Infectious Diseases*.

[5] White DA, Kiehn TE, Bondoc AY, Massarella SA (1999). Pulmonary nodule due to *Mycobacterium haemophilum* in an immunocompetent host. *American Journal of Respiratory and Critical Care Medicine*.

[6] Jang E-Y, Lee S-O, Choi S-H (2007). Case of pyomyositis due to *Mycobacterium haemophilum* in a renal transplant recipient. *Journal of Clinical Microbiology*.

[7] Elsayed S, Read R (2006). Mycobacterium haemophilum osteomyelitis: case report and review of the literature. *BMC Infectious Diseases*.

[8] Millar MJ, Bulliard C, Balachandran C, Maloof AJ (2007). *Mycobacterium hemophilum* infection presenting as filamentary keratopathy in an immunocompromised adult. *Cornea*.

[9] Modi D, Pyatetsky D, Edward DP (2007). *Mycobacterium haemophilum* a rare cause of endophthalmitis. *Retina*.

[10] Keller M, Mak A, Thibert L, Rene P, Klein MB (2008). Mycobacterium haemophilum epididymal abscess in a renal transplant patient. *Journal of Clinical Microbiology*.

[11] Phowthongkum P, Puengchitprapai A, Udomsantisook N, Tumwasorn S, Suankratay C (2008). Spindle cell pseudotumor of the brain associated with *Mycobacterium haemophilum* and *Mycobacterium simiae* mixed infection in a patient with AIDS: the first case report. *International Journal of Infectious Diseases*.

[12] Kay MK, Perti TR, Duchin JS (2011). Tattoo-associated *Mycobacterium haemophilum* skin infection in immunocompetent adult, 2009. *Emerging Infectious Diseases*.

[13] Hamsch C, Hartschuh W, Enk A, Flux K (2011). A chinese tattoo paint as a vector of atypical mycobacteria-outbreak in 7 patients in Germany. *Acta Dermato-Venereologica*.

[14] Wollina U (2011). Nodular skin reactions in eyebrow permanent makeup: two case reports and an infection by *Mycobacterium haemophilum*. *Journal of Cosmetic Dermatology*.

[15] Lindeboom JAH, Kuijper CF, Van Furth M (2007). Inguinal lymphadenitis caused by *Mycobacterium haemophilum* in an immunocompetent child. *Pediatric Infectious Disease Journal*.

[16] Whipps CM, Lieggi C, Wagner R (2012). Mycobacteriosis in zebrafish colonies. *ILAR Journal*.

[17] Hernandez-Divers SJ, Shearer D (2002). Pulmonary mycobacteriosis caused by *Mycobacterium haemophilum* and M marinum in a royal python. *Journal of the American Veterinary Medical Association*.

[18] Pai H-H, Chen WC, Peng CF (2003). Isolation of non-tuberculous mycobacteria from hospital cockroaches (*Periplaneta americana*). *Journal of Hospital Infection*.

[19] Jacob B, DeBey BM, Bradway D (2006). Spinal intradural *Mycobacterium haemophilum* granuloma in an American Bison (*Bison bison*). *Veterinary Pathology*.

[20] Saubolle MA, Kiehn TE, White MH, Rudinsky MF, Armstrong D (1996). *Mycobacterium haemophilum*: microbiology and expanding clinical and geographic spectra of disease in humans. *Clinical Microbiology Reviews*.

[21] Abbott MR, Smith DD (1980). The pathogenic effects of *Mycobacterium haemophilum* in immunosuppressed albino mice. *Journal of Medical Microbiology*.

[22] Lindeboom JA (2012). Surgical treatment for non tuberculous mycobacterium (NTM) cervicofacial lymhadenitis in children. *Journal of Oral and Maxillofacial Surgery*.

[23] Teh CL, Kong KO, Chong APY, Badsha H (2002). *Mycobacterium haemophilum* infection in an SLE patient on mycophenolate mofetil. *Lupus*.

[24] Aslam A, Green RL, Motta L, Ghrew M, Griffiths CE, Warren RB (2013). Cutaneous *Mycobacterium haemophilum* infection in a patient receiving infliximab for psoriasis. *British Journal of Dermatology*.

